# Selective ATP hydrolysis inhibition in F1Fo ATP synthase enhances radiosensitivity in non-small-cell lung cancer cells (A549)

**DOI:** 10.18632/oncotarget.18657

**Published:** 2017-06-27

**Authors:** Yupei Wang, Qinzheng Hou, Guoqing Xiao, Shifeng Yang, Cuixia Di, Jing Si, Rong Zhou, Yancheng Ye, Yanshan Zhang, Hong Zhang

**Affiliations:** ^1^ Institute of Modern Physics, Chinese Academy of Sciences, Lanzhou 730000, Gansu, China; ^2^ CAS Key Laboratory of Heavy Ion Radiation Biology and Medicine, Institute of Modern Physics, Lanzhou 730000, Gansu, China; ^3^ Gansu Wuwei Tumor Hospital, Department of Science and Technology, Wuwei 733000, Gansu, China; ^4^ Key Laboratory of Heavy Ion Radiation Medicine of Gansu Province, Institute of Modern Physics, Lanzhou 730000, Gansu, China; ^5^ University of Chinese Academy of Sciences, Beijing 100049, China; ^6^ College of Life Science, Northwest Normal University, Lanzhou 730070, Gansu, China; ^7^ School of Pharmacy, Lanzhou University, Lanzhou 730000, Gansu, China

**Keywords:** F1Fo-ATP synthase, X-ray radiation, radiosensitivity, mitochondrial membrane potential

## Abstract

**Background:**

F1Fo-ATP synthase (F1Fo-ATPase) is a reversibly rotary molecular machine whose dual functions of synthesizing or hydrolyzing ATP switch upon the condition of cell physiology. The robust ATP-hydrolyzing activity occurs in ischemia for maintaining the transmembrane proton motive force of mitochondria inner membrane, but the effect of F1Fo-ATPase on X-ray response of non-small-cell lung cancer (NSCLC) cells is unknown.

**Methods and Findings:**

We studied whether ATP hydrolysis affected X-ray radiation induced cell death. NSCLC cells (A549) were pretreated with BTB06584 (BTB), an elective ATP hydrolysis inhibitor, followed by X-ray radiation. Cell viability and clonogenic survival were markedly decreased, clear indications of enhanced radiosensitivity through BTB incubation. Additionally, ATP5α1 was upregulated in parallel with elevated ATP hydrolytic activity after X-ray radiation, showing an increased mitochondrial membrane potential (ΔΨm). ATP hydrolysis inhibition led to collapse of ΔΨm suggesting ATP hydrolytic activity could enhance ΔΨm after X-ray radiation. Furthermore, we also demonstrated that apoptosis was pronounced with the prolonged collapse of ΔΨm due to hydrolysis inhibition by BTB incubation.

**Conclusion:**

Overall, these findings supported that ATP hydrolysis inhibition could enhance the radiosensitivity in NSCLC cells (A549) after X-ray radiation, which was due to the collapse of ΔΨm.

## INTRODUCTION

Lung cancer is one of the worldwide common types of malignant tumors with two main categories: non-small cell lung cancer and small cell lung cancer [1]. Non-small-cell lung cancer (NSCLC) is responsible for over 75% of lung cancer in many countries [2–4]. Because the early onset of NSCLC is asymptomatic, it is often diagnosed in its late clinical stage with poor prognosis, metastasis and high fatality rate [4]. Taking into consideration that their resistance to radiation and drug treatment [4–6], it is necessary to find a target to improve the radiosensitivity.

F1Fo ATP synthase, or named ATPase (EC 3.6.1.3) (referred as the same enzyme hereafter), is an enzyme complex which presents in all organisms and predominantly locates on the inner membrane of mitochondria [7, 8]. F1 domain of F1Fo-ATP synthase makes clockwise rotation to catalyze the phosphorylation of ADP to ATP by exploiting the transmembrane proton motive force (Δp) [9]. However, when Δp collapse, F1 domain makes the counter clockwise rotation and switches its catalytic activity to ATP hydrolysis accompanied by transportation of H^+^ back to intermembrane space [10–12], which is usually associated with deprivation of oxygen or experiencing ischemia in numerous tissues [13, 14]. Increasing evidence highlight the role of the ATP synthase/hydrolase as key molecular and enzymatic switch between cell life and death [15]. ATP hydrolysis contributes to radioresistance for its ability to enhance mitochondrial membrane potential (ΔΨm) and ensures cell survival and proliferation after ionizing radiation, which has not yet been reported in cancer cells.

In cancer cells, because of metabolic and mitochondrial defects, cellular bioenergy is best characterized by predominant use of glycolysis to generate ATP instead of mitochondria even under aerobic conditions, which is proposed as Warburg effect [16, 17]. Even so, inhibited ATP synthase reduces ATP content, in turn ATP could be hydrolyzed by ATPase, pumping out a proton from mitochondrial matrix into the intermembrane space under glycolytic conditions [18–20]. It indicates ATP synthase not only participates in energy synthesis but also regulates ΔΨm in cancer cells. After radiation, changes of ΔΨm in A549 remain controversial in different time point and radiation dose [21, 22]. Oligomycin inhibits both F1Fo ATP synthase and ATPase activities resulting in blocking energy generation in normal tissue so it isn’t fit for application [23]. Choosing a selective inhibitor can explain the regulation mechanism of ATPase. A novel molecule BTB06584 (hereafter referred as BTB) developed by Ivanes et al. represents a valuable tool to selectively inhibit mitochondrial ATPase activity without compromising ATP synthase [24]. In this study, we for the first time confirmed ATPase played a role in enhancing ΔΨm after X-ray radiation and inhibition of ATP hydrolysis activity using a chemical inhibitor (BTB) could enhance the radiosensitivity of X-ray radiation through collapse of ΔΨm in A549.

## RESULTS

### Assessment of optimal concentration of BTB in NSCLC cells (A549)

**Figure 1:**
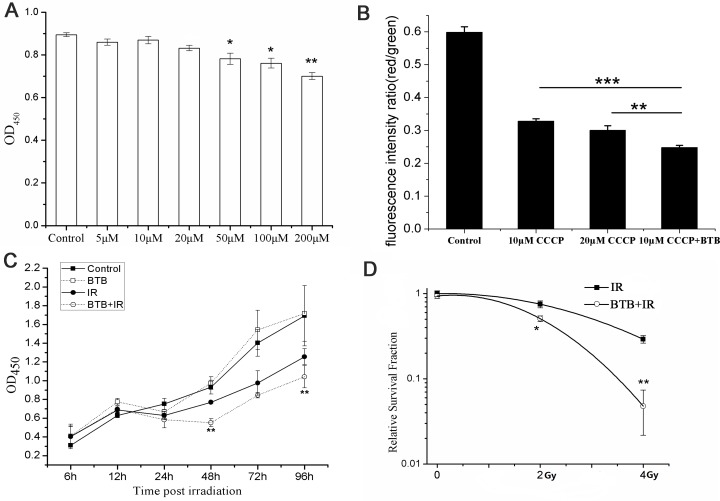
Combination of BTB with X-ray radiation (IR) increased radiation sensitivity in A549 by inhibiting F1Fo ATPase **(A)** Cells were incubated with 5-200 μM BTB. Cell viability was measured by CCK8 assay at 24 h after X-ray radiation. Multiple comparisons by one-way ANOVA with Dunnett post-hoc test. *p < 0.05, **p < 0.01. **(B)** ΔΨm was showed as the ratio of red to green fluorescence in each treatment. Data are mean ± SD of triplicate measurements. **P < 0.01, *** P < 0.001, t-test. **(C)** Cell proliferation was analyzed by CCK8 assay. Growth curve was showed for each treatment at 0, 6, 12, 24, 48, 72 and 96 h post X-ray radiation with or without 10μM BTB. Data are mean ± SD of triplicate measurements. BTB+IR compared to IR, *P < 0.05, ** P < 0.01, t-test. **(D)** Cell surviving fraction curve was performed with cell clonogenic survival assay. Cells were treated with or without BTB for 2 hours before radiation and plated for survival. The relative surviving fractions were calculated as surviving fraction of treated cells divided by that of the control. Results represent the mean±SD of triplicate experiments. *P < 0.05, ** P < 0.01, t-test.

Considering BTB could be a potential radiosensitizer in radiotherapy, we firstly assessed the cytotoxicity on several concentrations. The result showed cell survival decreased in a dose dependent manner from 50 to 200μM and had no significant difference under 50μM compared with control (Figure 1A). The effective concentration required for inhabiting ATPase need to be determined before further research. We used CCCP (10μM) to depolarize mitochondrial membrane potential as a negative control. BTB exacerbated the decrease in ΔΨm compared with negative control (Figure 1B). To minimize its toxicity on organism, 10μM BTB was chosen for further study.

### BTB contributes to radiosensitivity by inhibiting ATPase activity

Time course of cellular proliferation in four treatments IR(−)/BTB(−), IR(−)/BTB(+), IR(+)/BTB(−), IR(+)/BTB(+) were measured using CCK8 test (Figure 1C). After 4Gy X-ray irradiation(IR), the proliferation of IR(+)/BTB(+) treatment obviously decreased within 96h compared with IR(+)/BTB(−) treatment. Compared with control, BTB treatment had no significant difference in proliferation within 96h. This result suggested cell proliferation was suppressed at the presence of BTB after X-ray radiation.

The colony formation assay was performed to confirm whether ATPase activity inhibition by BTB contributed to increased radiosensitivity. The results showed that BTB treatment sensitized cells to radiation, by reducing the rate of colony formation from 29.13±2.97% to 4.78±2.6% at 4Gy X-ray radiation (Figure 1D). This result further suggested that BTB enhanced radiosensitivity by ATPase activity inhibition.

### Radiation enhanced ATP5α1 gene expression in mRNA and protein level

**Figure 2:**
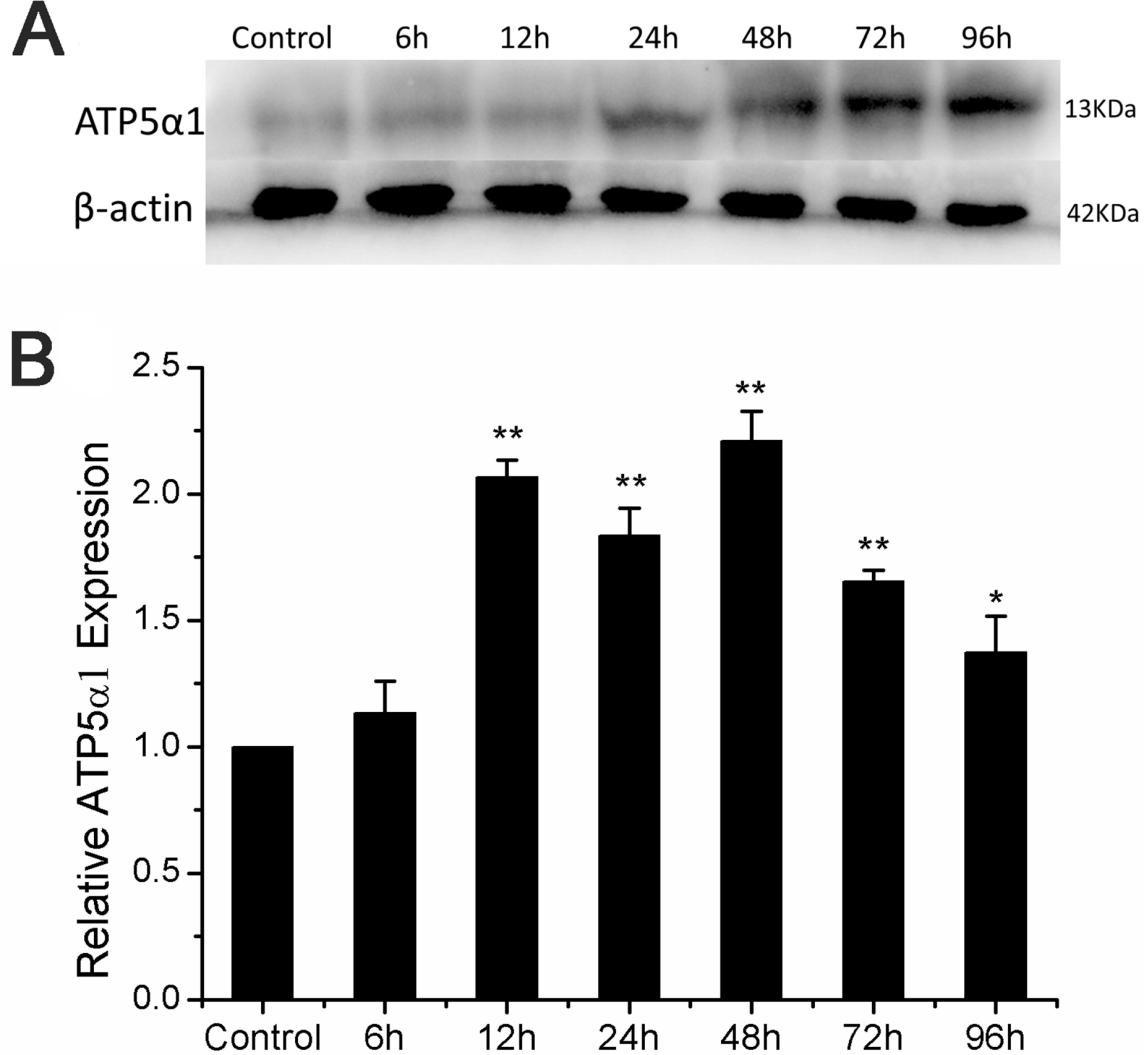
ATP5α1 expression altered in A549 after ionizing radiation **(A)** Upper panel showed protein expression level of ATP5α1 by western blot analysis. β-actin protein was used as an internal control. **(B)** ATP5α1 mRNA level expression was examined by semi quantitative RT-PCR and normalized by β-actin (calculated as 2^−ΔΔCT^) at time point of 6-96h after X-ray radiation. Histogram showed fold changes respect to control in mRNA expression. Results represent the mean±SD of triplicate measurements. Compared to control, *p< 0.05, **p< 0.01, t-test.

ATP5α1 is a subunit of F1Fo ATP synthase located in the catalytic domain F1, which is up regulation in several different human tumor samples, including breast cancer, hepatocellular carcinoma, colon cancer and prostate cancer involved in the progression and metastasis potential [25]. The levels of ATP synthase expression strongly correlated with large tumor size, poor tumor differentiation and advanced tumor stages [25]. These findings indicate that ATP synthase could be a potential radiation therapeutic target. As showed in the Figure 2B, ATP5α1 mRNA was overexpressed within 96h post radiation and more than two-fold expression was detected at 12h and 48h compared with control. Western blotting was used to further validate the level of ATP5α1 expression which was significantly increased during 24-96h post X-ray radiation compared with control (Figure 2A). These results indicated that X-ray radiation up regulated ATP5α1 expression within 96h post radiation.

### X-ray radiation enhanced mitochondrial F1Fo ATPase activity

**Figure 3:**
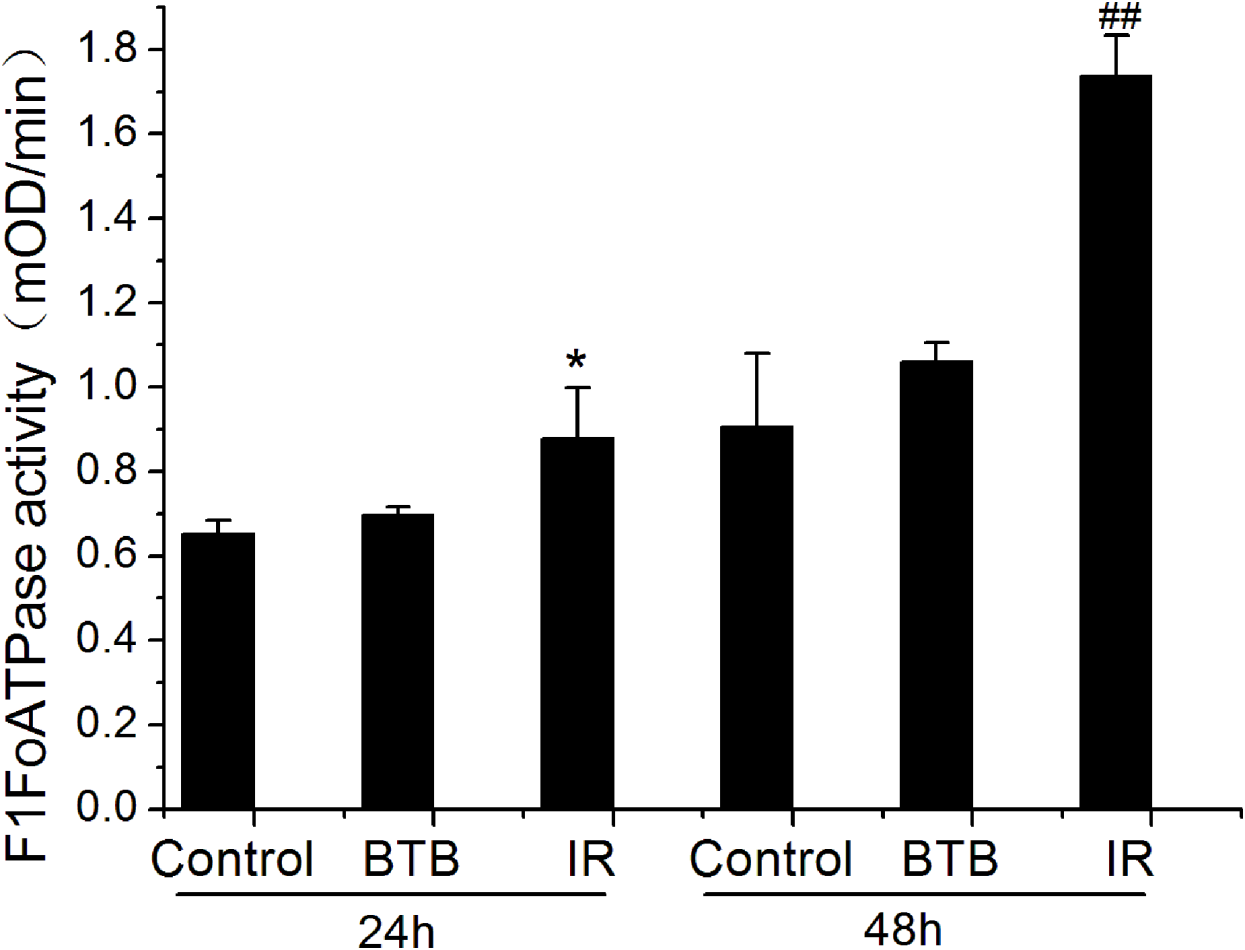
F1Fo-ATPase activity altered in A549 after X-ray radiation (IR) The rate of mOD changed with time (mOD/min) represented the F1Fo ATPase activity at 24h and 48h after IR. Results represent the mean±SD of triplicate measurements. *P< 0.5 compared with 24h control. ## P< 0.01 compared with 48h control, t-test.

Considering the Warburg effect in cancer cells, energy generation doesn’t predominantly depend on ATP synthase. The result of increased expression of ATP synthase is our primary focus. The mitochondria membrane protein was extracted for ATPase capacity detection using an ELISA kit. ATPase activity was significantly increased after X-ray radiation compared with control (Figure 3). Moreover, ATPase activity was further elevated at 48h post X-ray radiation. However, the activity of extracted ATPase (monomers) cannot be inhibited by addition of BTB, because the inhibition mechanism of BTB is related to conformation of ATPase dimers and oligomers on mitochondria membrane.

### X-ray radiation combined with BTB caused collapse of mitochondrial membrane potential (ΔΨm)

**Figure 4:**
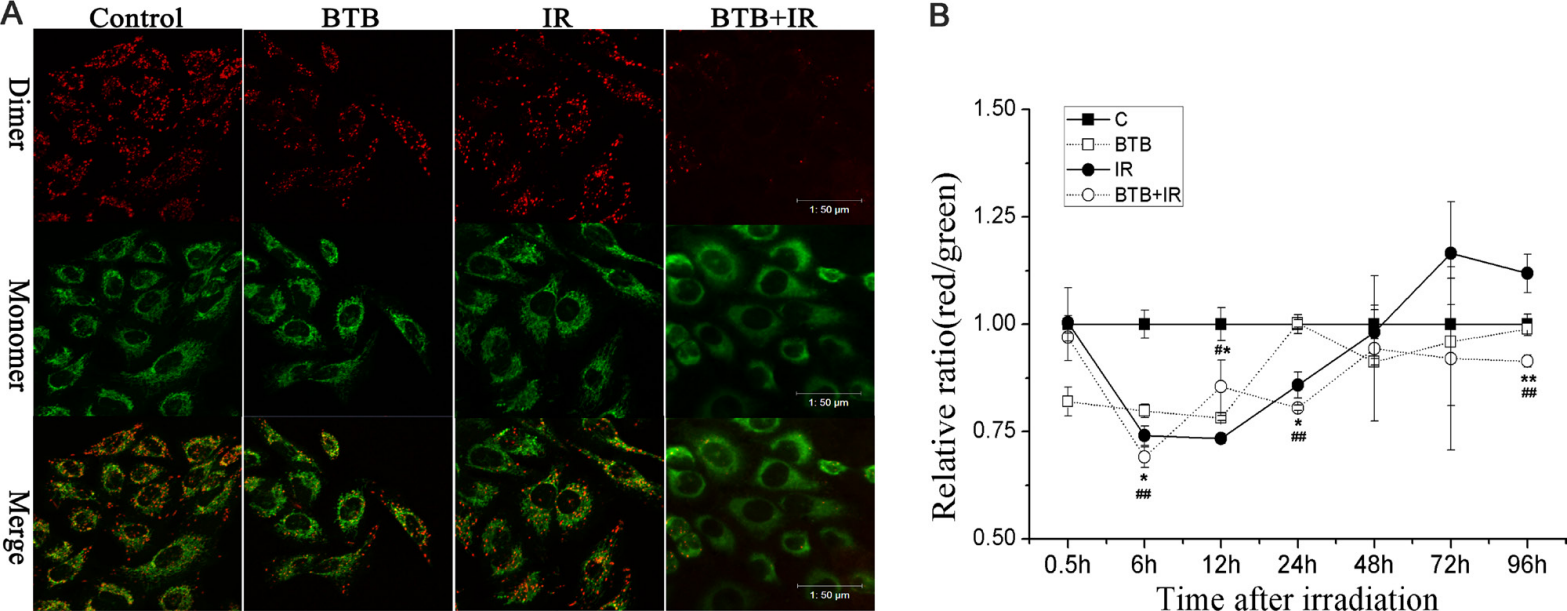
Changes of mitochondrial membrane potential (ΔΨm) **(A)** Representative micrographs captured by confocal laser scanning microscope were evaluated for ΔΨm by staining with JC-1 at 24h post ionizing radiation. Red dimers indicated normal mitochondrial function and green monomers indicated collapse of ΔΨm with cytoplasmic fluorescence. Scale bar = 50μm. **(B)** Kinetics of changes of ΔΨm was showed as the ratio of red to green fluorescence in each treatment staining with JC-1 at 0.5, 6, 12, 24, 48, 72 and 96 h after X-ray radiation (IR) with or without 10μM BTB treatment. Data are expressed as means±SD of triplicate measurements. IR compared with IR+BTB, *p< 0.05, **p< 0.01, t-test. Control compared with IR+BTB, #p< 0.05, ##p< 0.01, t-test.

BTB blocks H^+^ translocation to intermembrane space and recovery of transmembrane potential. To determine whether BTB incubation could inhibit mitochondrial membrane potential recovery after X-ray radiation, changes in ΔΨm was assessed by immunofluorescence using JC-1. As shown in (Figure 4A), compared with control, cells exposed to 4Gy X-ray radiation combined with BTB showed a marked reduction in red/green fluorescence intensity ratio at 24h post radiation, suggesting a loss of ΔΨm. BTB or radiation alone treatment showed no significant difference compared with control. The time course of ΔΨm was also quantified by the red/green fluorescence ratio using plate reader (Figure 4B). The result showed that ΔΨm was initially decreased in 0.5-6h in all treated groups and then began to recover. ΔΨm of radiation treatment was increased over basal level from 48h post-irradiation. ΔΨm of BTB treatment was also recovered to basal level at 96h. However, BTB combined with X-ray radiation treatment maintained a lower ΔΨm compared with control and radiation group.

### The BTB enhanced cell apoptosis after X-ray radiation

**Figure 5:**
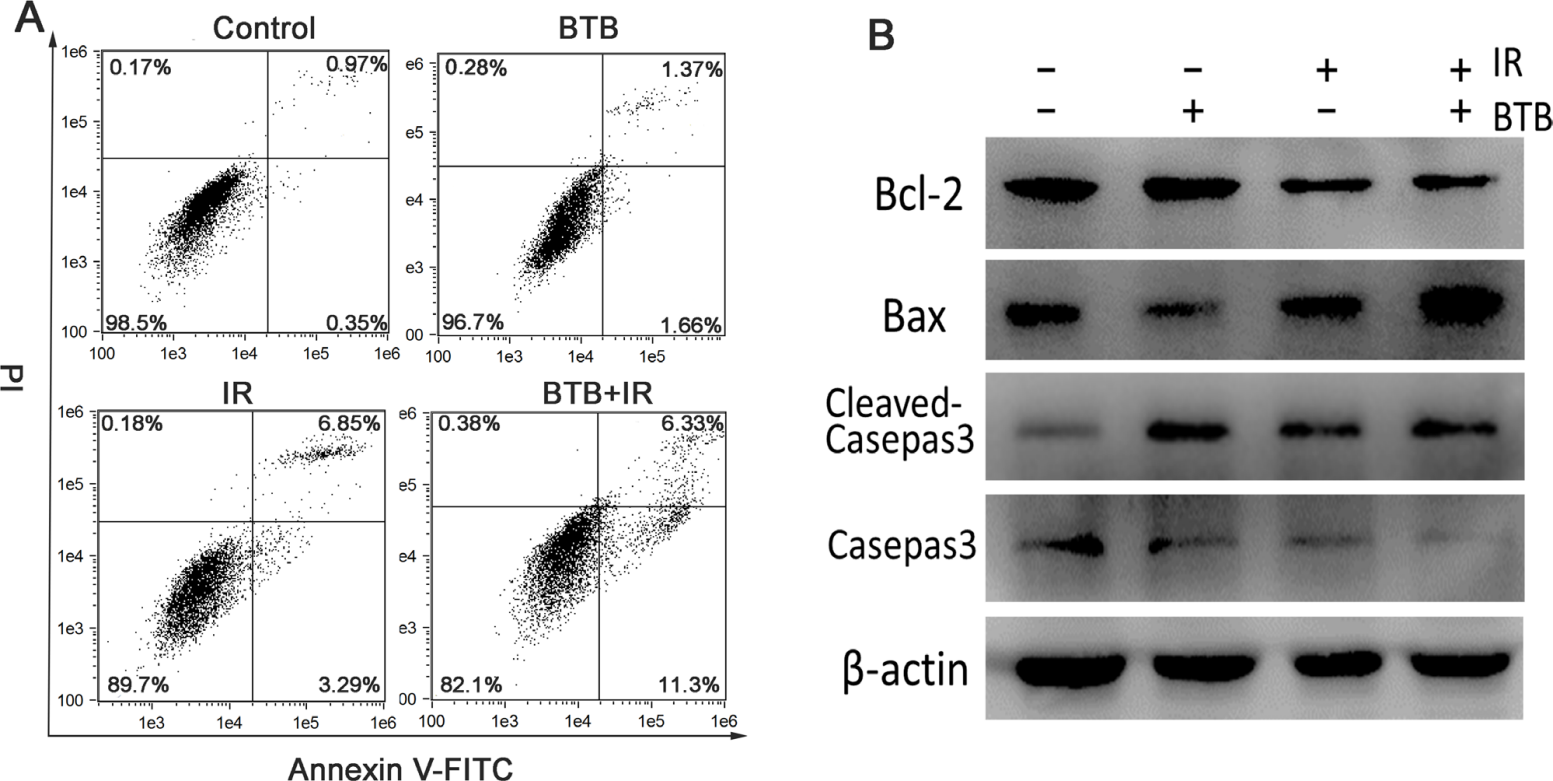
Combination BTB with ionizing radiation increased the apoptosis rate in NSCLC cells **(A)** Cell apoptosis was detected using flow cytometry analysis. Apoptosis was analyzed by co-staining with annexin V-FITC and PI. The visual images of each call in control were used to set the threshold gating to determine the percentage of viable cells, early/late apoptotic cells and necrotic cells. The cell apoptosis percentages show early (annexin V+and PI–) and late (annexin V+ and PI+) apoptotic cells. **(B)** Expression of apoptotic related protein, including Bcl2, Casepas3, Bax were detected by western blot analysis for identifying apoptotic mechanisms. β-actin protein was used as an internal control.

The increased radiosensitivity results in more cell death, which is likely caused by apoptosis. We therefore used flow cytometry to detect cellular apoptosis. The cells were dual-stained with annexin V and PI. As shown in (Figure 5A), cells treated with BTB combined 4Gy radiation showed increased early apoptosis compared with radiation alone (above three fold). Compared with the control, there was a slight increase of apoptosis rate in BTB treated alone (increased by 1.71%). The data demonstrated that BTB combined X-ray radiation induced more apoptosis than X-ray radiation treated alone (increased by 7.81%). Since BTB pretreatment had a marked response on X-ray irradiation-induced apoptosis, the possible molecular mechanisms were further explored. X-ray-radiation-induced apoptosis involves the Bcl-2 family and is mediated by extrinsic and intrinsic pathways [26]. Bax is a pro-apoptotic member in the Bcl-2-family with the ability of mitochondrial outer membrane permeabilization and consequently promoting the release of cytochrome c, whereas anti-apoptotic members such as Bcl-2 work as protectors of the outer membrane and preserve its integrity by opposing Bax function [27]. The increased apoptosis in BTB incubation was accompanied by downregulation of Bcl-2, upregulation of Bax after X-ray radiation (Figure 5B), and therefore an increase of Bax/Bcl-2 ratio. We further examined the possible involvement of caspase cascade by assessing the expression of Caspase 3, a key executor of apoptosis protein, which was activated by the treatment of BTB combined X-ray radiation (Figure 5B).

## DISCUSSION

Based on asymptomatic oncogenesis and poor prognosis in NSCLC patients, external beam radiotherapy was conventional therapeutic approach to patients with metastatic or relapsed NSCLC. With the purpose of deliver more accurate treatment to tumors whilst sparing radiation dosage to normal tissues, advanced technology have been emerged in thoracic radiotherapy, but the 5-year rate was still under 20% in NSCLC [28]. The greatest potential to increase the effectiveness of radiotherapy lies in combining it with radiosensitizer, either by targeting tumor cells directly or by modifying the tumor microenviroment [29]. As selective inhibition of ATPase not only has no effect on ATP synthesis, but also blocks ΔΨm recovery, the F1Fo-ATP synthase with hydrolysis activity becomes a potential target for increasing cancer cell death after X-ray radiation without inducing side effects on normal cells.

BTB was introduced to combine with X-ray radiation for its specific inhibition of ATP hydrolysis activity for the first time. Ivanes et al reported that BTB had no effect on ΔΨm or O_2_ consumption in Hela and HL-1 cells and didn’t affect energy metabolism on aerobic condition by zebrafish embryo toxicity test [24]. In our work, we didn’t find the significant changes in cell morphology, cell proliferation and ATP content induced by BTB exposure. The inhibition function of BTB depends on inhibitor protein of F1 subunit (IF1), the endogenous inhibitor of the F1Fo-ATPase activity expressed in various types of human cancers [30]. The mimic IF1 activity is useful in mitochondrial physiology [31]. Evidence showed that ATPase activity was sufficient to maintain ΔΨm in CCCP(5μM)-treated cells at expense of cellular ATP and IF1 blocked ATPase function, thus promoting collapse of ΔΨm [32]. We showed that addition of BTB to 10μM CCCP-pretreated cells also exacerbated the depolarization of ΔΨm, which was even lower than 20μM CCCP treated cells, indicating an efficient inhibition ability to ATPase. Most lung cancer cell types including A549 are PET (positron emission tomography) positive with higher glycolytic rate for ATP generation, which definitely means a Warburg effect in A549. The function of F1Fo ATP synthase in mitochondria respiratory chain (MRC) remains largely enigmatic in cancers. Our results showed that combination of BTB with X-ray enhanced the radiosensitivity by reduced cell proliferation and clone survival rate which was closely related to F1Fo ATP synthase.

We found BTB didn’t cause significant increase in ATP content compared with control suggesting F1Fo ATP synthase didn’t act as an ATPase on normal condition (data not show). When glycolysis was inhibited, a significant increase in A549 cells’ oxygen consumption rate was detected as compensatory mechanism via increase of mitochondrial respiration which suggested a healthy F1Fo ATP synthase in mitochondria inner membrane [33]. Developing Warburg effect emphasizes while glycolysis is indeed drastically upregulated in almost all cancer cells, mitochondrial respiration continues to operate normally at rates proportional to oxygen supply [34]. Exposure of cancer cells in respiration inhibitors blocks proliferation *in vitro* and inhibition of respiration suppresses tumor growth in xenografts *in vivo*, which argue that mitochondrial respiration is essential for rapid proliferation [35]. We assumed that mitochondrial respiration and F1Fo ATP synthase contributed to cancer cell proliferation on aerobic condition. ATP hydrolysis activity was initiated by X-ray radiation which was supported by elevated hydrolytic activity of F1Fo ATPase and over-expressed ATP5α1 (α subunit in F1Fo ATP synthase) after X-ray radiation, therefore, giving a chance to BTB to disrupt this adaptive response. However, as the measurement of ATP synthase activity is out of mitochondria environment, it is difficult for maintaining the inhibition conformation through BTB. Thus the direct evidence for inhibiting ATP hydrolysis is inaccessible in our current experimental set.

Unlike acute ischemia in myocardial cell, cancer cells always grow in hypoxia regions with depressed oxidative phosphorylation and still manage to maintain a normal ΔΨm [36]. It has been reported that in A549 cells glycolytic or ATP synthase inhibition depolarized mitochondria after respiratory inhibition [37]. The depolarization not happened in macrophages with complete inhibition of respiration, instead, observed a high ΔΨm [19]. Respiration and ATP hydrolysis were each individually sufficient to maintain ΔΨm in HepG2 cells [38]. Therefore, when respiration is inhibited, the maintaining of ΔΨm depends on ATPase and available ATP. These evidences supported that F1Fo-ATP synthase hydrolyzed a significant proportion of ATP in absence of respiratory companied by pumping H^+^ in mitochondrial inner membrane in order to maintain of ΔΨm in cancer cell. It has been clearly demonstrated that radiation induced oxidative stress and DNA damage promote mitochondrial outer membrane permeabilisation, loss of the ΔΨm (due to the permeabilisation of mitochondrial inner membrane) and ΔΨm-dependent transports [39]. It is reasonable to speculate F1Fo ATPase plays an important role in maintaining ΔΨm after X-ray radiation in A549, which contributes to cell survival and proliferation. As depolarized mitochondria membrane potential was a characteristic marker of irreversible cell apoptosis, we proposed that BTB blocked ΔΨm recovery by inhibiting ATP hydrolysis after X-ray radiation which was verified by JC-1 test. ΔΨm was decreased in first 12h in all treated groups. After that, interestingly, ΔΨm showed different level of increase, especially in X-ray radiation group. Increased ΔΨm were also observed in human K562 and HL60 cells about 24 h later after 12Gy of X irradiation [40]. Besides, 10Gy X-ray radiation increased mitochondrial respiration and contributed to hyperpolarization of ΔΨm in A549 [22]. Based on our results, the increased expression of ATP5α1 and activity of F1Fo ATPase at 24h are partly responsible for the observed hyperpolarization. However, increased ΔΨm was blocked by BTB treated group leading to a lower ΔΨm than basal level within 96h.

We also demonstrated that apoptosis was pronounced with the prolonged collapse of ΔΨm induced by BTB incubation. Loss of ΔΨm results in the release of various molecules that are usually confined to the intermembrane space of the mitochondria. Such molecules include enzymes called pro-caspases, cytochrome c and apoptosis inducing factor [41]. Anti-apoptotic (such as Bcl-2, Bcl-XL, Bcl-W and Mcl-1) and proapoptotic members (such as Bax, Bak and Bok) could regulate this process. In this study, Bax was markedly upregulated in BTB (+)/IR (+) group, which leading to mitochondrial outer membrane permeabilisation and consequently promoting the release of cytochrome c. When cytochrome c is released into the cytoplasm, it forms a complex polymer with caspase-9, apoptosis-activating factor 1 (Apaf-1), and resulting in a caspase cascade reaction. As an executioner caspase, full-length Caspases 3 exist as inactive proenzymes that undergo proteolytic processing at conserved aspartic residues to produce two fragments, large and small, that dimerize to form the active enzyme. Then, cleaved-caspase-3 executed apoptosis. Although elevated apoptosis was induced by decreased ΔΨm which is dependent on mitochondrial pathway activation and ROS production [21], the mechanism of cell death is more complex. Necrosis-like cell death can also cause decreased ΔΨm without activating other apoptosis-associated protein [42]. Most of respiration inhibitors could activate mitophagy through dissipation of the ΔΨm [43]. Basit et al. reported inhibition of complex I of the mitochondrial respiratory chain depolarized the ΔΨm, paralleled by increased opening of mitochondrial permeability transition pore, that stimulate autophagosome formation, mitophagy and an associated ROS increase, leading to activation of combined necroptotic/ferroptotic cell death [44]. However, some inhibitors induced a relatively limited decrease in ΔΨm that is rapidly balanced by the reverse hydrolysis activity of the ATPase [45]. So preventing the compensatory mechanism of hydrolysis activity facilitates mitophagy which can be achieved by addition of BTB. Lefebvre et al. reported following prolonged loss of ΔΨm, IF1 was essential for mitophagy in cultured cells [32]. This condition is similar as addition of BTB in radiation treated cell. In some settings, autophagy and apoptosis seem to be interconnected positively or negatively, introducing the concept of ‘molecular switches’ between them [46]. Therefore, it is necessary to study the F1Fo-ATP synthase related cell death more intensively in further studies.

In summary, we demonstrated that the reverse operation of F1Fo-ATPsynthase contributed to maintain the ΔΨm after X-ray radiation. Meanwhile, inhibiting ATP hydrolysis by BTB led to collapse of ΔΨm, which was important to sensitize the cancer cells. The finding of the present study provided a novel inhibition target for enhancing radiosensitivity and also proposed that BTB could be a potential radiosensitizer in radiation therapy.

## MATERIALS AND METHODS

### Cell culture and treatment

NSCLC cells (A549) were purchased from the China Center for Type Culture Collection (CCTCC) (Wuhan, China) and cultured in RPMI1640 medium (HyClone™, USA) supplemented with 1 % penicillin/streptomycin (Gibco®, USA) and 10 % heat-inactivated fetal bovine serum (FBS; HyClone™, USA). Cells were incubated at 37°C in a humidified atmosphere containing 5 % CO_2_.

### Exposure of cells to drugs and radiation

Cells in exponential growth were seeded onto culture dishes and were cultured overnight to allow adherence to the dish. Inhibitor BTB was purchased from Selleck (USA) and dissolved in dimethyl sulfoxide (DMSO; Sigma-Aldrich, USA) at 50mM concentration and further diluted into growth medium at end concentration. Cells were preincubated with BTB (at a final concentration of 10 μM) for 2 hours before radiation ensuring absorption of drug. Radiation exposures were performed using an X-ray machine (Faxitron RX-650, USA) with 100 kVp at a distance of 45cm from the focus. The dose rates for delivering were 0.32 Gy/min. Subsequently, cells were cultured in the presence of the BTB for 6–72 h before analyses. For determining the effective concentration of BTB, cells were first exposed to a mitochondrial proton uncoupler CCCP (10 μM or 20μM) for 1h resulting in a reversal of the activity of the F1Fo-ATP synthase which maintain ΔΨ at the expense of cellular ATP. Then CCCP was replaced with BTB for 2h. Effective inhibition of the ATPase will then cause a severe loss of ΔΨm.

### Cell proliferation analysis

Cell proliferation was analyzed using Cell Counting Kit-8(CCK-8) (Dojindo, Japan) following the manufacturer’s recommendation. Briefly, 100μL of cell suspension was inoculated in 96-wells plate (2000 cells/well) and incubated for 6, 12, 24, 48, and 72 hours. Then 10μL CCK8 was added to each well for 2 hour, followed by light absorbance measurement at a wavelength of 450 nm using a microplate reader(Tecan infinite 200 M).

### Mitochondrial membrane potential (ΔΨm) assay

The change in the mitochondrial membrane potential (ΔΨm) was assessed using JC-10 mitochondrial membrane potential assay kit (Solarbio, Cat. CA1310) following instructions provided by the manufacturer. This dye reagent, 5,5′,6,6′-tetrachloro-1,1′,3,3′-tetraethylbenzimidazolylcarbocyanine iodide (JC-1), spontaneously forms complexes known as J-aggregates with intense red fluorescence in healthy cells with high ΔΨm. In apoptotic or unhealthy cells, however, the ΔΨm collapses and JC-1 no longer accumulates within the mitochondria and remains in the monomeric form, which yields only green fluorescence. Cell suspension treated with BTB or CCCP were incubated with JC-1 working solution for 20min at 37°C and selected by centrifugation at 600g for 4 min. After washing twice with staining buffer, cells were suspended with staining buffer again and transferred in to white 96 wells plate (100μL/well). The cellular fluorescence intensity of both JC-1 green monomers (excitation 485 nm, emission of 530 nm) and red dimers (excitation 530 nm, emission 590 nm) were measured on the microscopemicroplate reader (Tecan infinite 200M). The results are showed as a ratio of red dimers to green monomers fluorescence in relation to the control fluorescence ratio. Images of JC-1 stain were observed under a confocal microscope equipped with a digital camera (LSM700; Carl Zeiss).

### Colony formation assay

Immediately after treated, cells were trypsinzed and resuspended. Suspensions were diluted and the proper number of cells was seeded in 60-mm dishes. After incubation for 10–14 days, colonies were fixed with methanol and stained with crystal violet. Colonies containing more than 50 cells were scored as surviving cells. Images of air-dried plates were counted with naked eyes. Plating efficiency (PE) at each dose was calculated as number of colonies divided by number of cells plated in control. The surviving fraction (SF) was calculated as treated group respected to the PE of the non-irradiated control and plotted at each physical dose. Survival curves were fitted using the linear-quadratic. Each experiment was performed at least three times.

### Real-time PCR

Total RNA was extracted from the cultured cells using the TaKaRa MiniBEST Universal RNA Extraction Kit (Takara, Japan) and the concentration of extracted RNA was estimated by optical density measurement (A260/A280 ratio) with a BioPhotometer (Eppendrof, Genmany). The purified RNA was used to generate cDNA using the Prime Script RT reagent kit (Takara, Japan). Real-time quantitative PCR analysis of ATP5α1 and the reference gene GAPDH was carried out using the one step SYBR PrimeScript kit (Takara, Japan) following the manufacturer’s specifications. Primers were designed as follow. ATP5α1: forward: 5′-tgtaaaacgacggccagtGGCCTATCCTGGTGATGTG-3′ reverse: 5′-caggaaacagctatgaccGCTTCATAGCCCTGGTTTGG-3′. GAPDH: forward: 5′-GAAGGTGAAGGTCGGAGTC-3′ reverse: GAAGATGGTGATGGGATTTC. The fold change of mRNA expression was normalized to β-actin using the 2^−ΔΔCt^ quantification approach.

### Western blot assay

Whole protein lysates were extracted from the cells with RIPA lysis buffer (Solarbio, China) added PMSF and quantified by BCA Protein Assay Kit (Pierce, Rockford, IL, USA). Equal amounts of protein were fractionated on 10% SDS-PAGE. Western blotting was performed as previously described. The primary antibodies used were as follows: mouse anti-α-F1-ATPase (Abcom, Cambridge, MA), rabbit monoclonal anti-Bax (ImmunoWay, USA), anti-Bcl2 (GeneTax, USA), anti-caspase3 (Signaling Technology, USA) and anti-β-actin (Signaling Technology, USA) antibodies (1:500). HRP-linked anti-mouse or anti-rabbit IgG antibodies (Zsjqbio, China) were used as secondary antibodies (1:10000). The antigens were visualized by the electrochemiluminescence (Millipore, Darmstadt, Germany). The results were normalized by β-actin levels.

### ATP synthase activity measurement

ATP synthase activity was determined by a commercially available ATP Synthase Enzyme Activity Microplate Assay Kit (Abcam, Cambridge, MA) according to the manufacturer’s instructions. The kit is designed to immunocapture ATP synthase proteins within the wells. The immunocaptured ATP synthase enzyme operates in the “reverse” direction as the physiological role of ATP synthase: it hydrolyzes ATP to ADP and phosphate. This production of ADP is ultimately coupled to the oxidation of NADH to NAD+ which is monitored as a decrease in absorbance at 340 nm. The change in absorbance at 340 nm between 12 and 30 minutes were record. Rate is calculated as ΔOD (m)/ΔT(min).

### Apoptosis assays

Apoptotic cells were quantified by Annexin V-FITC/Propidium iodide (PI) double staining. Cells from different treatment groups were incubated for 24 h and were harvested with trypsin. Cells were washed twice with PBS and diluted with binding buffer to a concentration of 1×10^5^ cells/ml. Then 5 μl annexin V and 5μl propidium iodide (Annexin V-FITC Apoptosis Detection Kit I, BD, USA) were added to 150ul of cell suspension, which was then incubated in the dark for 15 min at room temperature. Fluorescence of the cells was immediately analyzed on a flow cytometer FlowSight (Amnis, Seattle, WA, USA). In the annexin V/PI quadrant gating, annexin V (−)/PI (−), annexin V (+)/PI (−), and annexin V (+)/PI (+) were used to identify the fraction of viable cells, early apoptotic cells, and late apoptotic/necrotic cells, respectively.

### Statistical analysis

Data are shown as means ± SD. Student’s t-tests and one-way ANOVA with Dunnett post-hoc test were used to detect differences between groups; *p < 0.05 and **p < 0.01 considered statistically significant. All statistical calculations were performed using the SSPS19.0 software.
